# NASH triggers cardiometabolic HFpEF in aging mice

**DOI:** 10.1007/s11357-024-01153-9

**Published:** 2024-04-17

**Authors:** Dániel Kucsera, Mihály Ruppert, Nabil V. Sayour, Viktória E. Tóth, Tamás Kovács, Zsombor I. Hegedűs, Zsófia Onódi, Alexandra Fábián, Attila Kovács, Tamás Radovits, Béla Merkely, Pál Pacher, Péter Ferdinandy, Zoltán V. Varga

**Affiliations:** 1 Department of Pharmacology and Pharmacotherapy, Semmelweis University, Budapest, Hungary; 2 HCEMM-SU Cardiometabolic Immunology Research Group, Budapest, Hungary; 3 MTA-SE Momentum Cardio-Oncology and Cardioimmunology Research Group, Budapest, Hungary; 4 Heart and Vascular Center, Semmelweis University, Budapest, Hungary; 5 Laboratory of Cardiovascular Physiology and Tissue Injury, National Institutes of Health/National Institute On Alcohol Abuse and Alcoholism, Bethesda, MD USA; 6 Pharmahungary Group, Szeged, Hungary; 7 Department of Surgical Research and Techniques, Semmelweis University, Budapest, Hungary

**Keywords:** Metabolic dysfunction, Inflammation, Fatty liver, Strain rate analysis, Liver fibrosis

## Abstract

Both heart failure with preserved ejection fraction (HFpEF) and non-alcoholic fatty liver disease (NAFLD) develop due to metabolic dysregulation, has similar risk factors (e.g., insulin resistance, systemic inflammation) and are unresolved clinical challenges. Therefore, the potential link between the two disease is important to study. We aimed to evaluate whether NASH is an independent factor of cardiac dysfunction and to investigate the age dependent effects of NASH on cardiac function. C57Bl/6 J middle aged (10 months old) and aged mice (24 months old) were fed either control or choline deficient (CDAA) diet for 8 weeks. Before termination, echocardiography was performed. Upon termination, organ samples were isolated for histological and molecular analysis. CDAA diet led to the development of NASH in both age groups, without inducing weight gain, allowing to study the direct effect of NASH on cardiac function. Mice with NASH developed hepatomegaly, fibrosis, and inflammation. Aged animals had increased heart weight. Conventional echocardiography revealed normal systolic function in all cohorts, while increased left ventricular volumes in aged mice. Two-dimensional speckle tracking echocardiography showed subtle systolic and diastolic deterioration in aged mice with NASH. Histologic analyses of cardiac samples showed increased cross-sectional area, pronounced fibrosis and *Col1a1* gene expression, and elevated intracardiac CD68^+^ macrophage count with increased *Il1b* expression. Conventional echocardiography failed to reveal subtle change in myocardial function; however, 2D speckle tracking echocardiography was able to identify diastolic deterioration. NASH had greater impact on aged animals resulting in cardiac hypertrophy, fibrosis, and inflammation.

**Supplementary Information:**

The online version contains supplementary material available at 10.1007/s11357-024-01153-9.

## Introduction

The ever-growing burden of chronic cardiometabolic diseases, such as obesity, type 2 diabetes, hypertension, dyslipidemia, metabolic syndrome, systemic inflammation, and aging of the population, demands urgent resolution to these socio-economic and healthcare problems. Advanced stages of metabolic and cardiovascular diseases, such as non-alcoholic steatohepatitis (NASH) and heart failure with preserved and reduced ejection fraction (HFpEF and HFrEF), are leading causes of death worldwide [1, 2], with limited pharmacotherapeutic options.

Clinical observations suggest a potential interplay between non-alcoholic fatty liver disease (NAFLD), a progressive, chronic liver pathology, and heart failure with preserved ejection fraction (HFpEF), a complex syndrome with features of diastolic dysfunction, cardiac hypertrophy, fibrosis, enlarged atria [3–10]; however, a direct causal link between the two entity has not been established.

Both NASH and HFpEF are diseases with a large, heterogeneous population with coinciding comorbidities such as hypertension, diabetes, dyslipidemia, obesity, metabolic syndrome, and atrial fibrillation. Chronic systemic aged-dependent inflammation contributes to both diseases [11, 12]. There is a possibility that NASH, a meta-inflammatory stage of NAFLD, itself might inflict damage on the heart, but with so many overlapping factors, it is hard to determine whether mediators of NASH or the systemic burden of the co-morbidities fuels this link. As such, we aimed, in this study, to investigate the cardiac effects of NASH in middle-aged and aged mice without the systemic burden of obesity, insulin resistance, and hypertension.

## Materials and methods

### Experimental animals, diets, and ethical approval

Eight-week-old C57Bl/6 J male mice were purchased from Oncological Research Center, Department of Experimental Pharmacology, Budapest, Hungary. Two-four mice were housed per each individually ventilated cage, and were maintained under 12–12 light–dark cycle under appropriate conditions (20–24 °C and 35–75% relative humidity). Standard chow diet and tap water were available ad libitum.

Control (CON, E 15668–04) diet and Choline Deficient L-Amino Acid defined (CDAA, E15666–94) diet were purchased from SSNIFF GmbH (Soest, Germany). In short, CDAA diet is composed of crystalline amino acids with no choline and low methionine, and 1% cholesterol content. The energy intake is comprised by 31 kJ% of fats, 58 kJ% of carbohydrates, and 11 kJ% of proteins.

All experimental procedures were done in accordance with the Guide for Care and Use of Laboratory Animals published by US National Institutes of Health (NIH publication No. 85–23, revised 1996), with the EU Directive (2010/63/EU), and were approved by the National Scientific Ethical Committee on Animal Experimentation (PE/EA/1912–7/2017, Budapest, Hungary) and in compliance with the ARRIVE guidelines [13].

### Non-alcoholic steatohepatitis model

Twenty mice were aged up to 10 months (middle-aged cohort) and twenty more mice were aged up to 24 months (aged cohort). Male mice were used due to their greater propensity to frailty-driven cardiac decline. In our previous study, we observed that male mice with NASH developed greater fibrosis compared to females [14]. Additionally, liver fibrosis was identified as an elevated risk for mortality in patients with HFpEF [7], further supporting the choice of sex in our study.

At the start of the experiment, mice were randomized by their bodyweight. Mice were fed with either control or choline deficient diet for 8 weeks. On the 7^th^ week, experimental animals underwent conventional and 2D speckle tracking echocardiography. Following termination, organ and serum samples were collected and stored. Although patients either with NASH or HFpEF are mostly obese, the increased adiposity burdens both the liver and the cardiovascular system, and, furthermore, contributes to systemic inflammation by triggering the innate immune system.

Our aim in this study was to study the sole effects of NASH on the cardiac function; thus, we chose the CDAA diet, a diet that lacks adipogenic potential, because we wished to exclude the burden of obesity.

### Echocardiography

Anesthesia was induced with 5% -, and was maintained with 2% isoflurane. Cardiac functions were analyzed with the Vevo 3100 high-resolution in vivo echocardiograph (Fujifilm VisualSonics, Toronto, Canada) with a MX400 transducer. Two-dimensional images were assessed by long-axis view for left ventricular volumes, and short-axis view for left ventricular diameter and wall thickness. Diastolic parameters were measured in apical four-chamber view. Early transmitral flow velocity (*E*) and septal mitral annular early diastolic velocity (*e′*) was measured with pulse wave and tissue Doppler, respectively.

Ejection fraction was calculated as [(LVEDV − LVESV)/LVEDV × 100]. Fractional shortening was calculated with the following formula: [(LVIDd − LVIDs)/LVIDd] × 100. Stroke volume (SV) was obtained as LVEDV − LVESV. Cardiac output was determined as SV × HR/1000. Left ventricular mass was calculated as {[(LVIDd + AWTd + PWTd)^3^ – LVIDd^3^] × 1.0} × 0.8 + 0.14.

Echocardiographic recordings were evaluated with the VevoLab software by a blinded operator.

#### Strain analysis with 2D speckle tracking

Two-dimensional speckle tracking echocardiography was performed to investigate myocardial strain and strain rate. These parameters enable us to study deformation of the longitudinal and circumferential cardiac myofibers. Long- and short-axis views of the left ventricle were acquired as described above. The recordings were exported to an offline workstation and were analyzed with the 2D Cardiac Performance Analysis v1.2 software (TomTec Imaging Systems, Unterschleissheim, Germany). The analysis procedure was performed by an operator blinded to the study groups.

Three cardiac cycles were used to quantify global longitudinal strain (GLS). To quantify global circumferential strain (GCS) and early diastolic strain rate (SrE), short-axis recordings were used with the same settings. Endocardial border was delineated manually; then, the software divided the left ventricle into six segments and tracked them. If low endocardial tracking fidelity was observed, the operator realigned the contour, and the calculation was repeated maximum three times. Systolic strains, and early diastolic strain rates of the 6 left ventricular segments were averaged over the three cardiac cycles, and were used to calculate GLS, GCS, SrE values. E/SrE was calculated using the E waves assessed by pulse-wave Doppler.

### Histologic analysis

Heart and liver samples were fixed in neutral buffered formalin for 24 h, then dehydrated and embedded in paraffin. Four µm thick sections were cut with microtome. All staining was imaged with Leica LMD6 microscope (Wetzlar, Germany) and with Leica DMI8 confocal microscope (Wetzlar, Germany).

#### Hematoxylin and eosin staining

Liver and cardiac tissues were deparaffinized, hydrated, and then stained with hematoxylin and counterstained with eosin.

#### Picrosirius-red staining

Heart and liver sections were stained with 0.0125% picrosirius-red for 1 h, then washed with 1% acetic acid. The level of fibrosis was quantified by the ImageJ software.

#### Lectin histochemistry

Heart sections were co-stained overnight at 4 °C for isolectin B4 and for wheat germ agglutinin with lectins conjugated with fluorescein isothocyanate and DyLight 594, respectively. After three washing steps, nuclei were labeled with DAPI. Subsequent washing steps were followed by coverslip mounting with Prolong® Gold Antifade Reagent (CST 9071S, Cell Signaling Technology, USA). Images were obtained with Leica LMD6 microscope (Wetzler, Germany). For further detail about the antibodies, please see Supplementary Table 1.

#### Immunohistochemistry

Antigens were retrieved in an acidic environment (citrate buffer pH = 6) for 15 min. Specimens were blocked with 3% H_2_O_2_ for 10 min and, subsequently, with 2.5% goat bovine serum albumin (9998S, Cell Signaling Technology, USA) for 1 h for endogenous peroxidases and for off-target antigens, respectively. Primary antibody for Iba1 (in 2.5% goat serum) was incubated overnight at 4 °C. Sections were washed three times with PBS, then the specimens were incubated with anti-rabbit IgG secondary antibody, then were washed and signals were developed with diaminobenzidine (ImmPact DAB EqV Peroxidase (HRP) Subrate, Vector Laboratories, Burlingame, CA, USA). For further details about the antibodies please see Supplementary Table 1.

#### qRT-PCR

Total RNA was isolated from snap frozen liver and cardiac samples by using the chloroform/isopropanol precipitation method. Reverse transcription from 1 µg of total RNA was performed to obtain cDNA with a Sensifast cDNA synthesis kit (Bioline, London, UK). SensiFAST SYBR Green master mix (Bioline, UK) was used to amplify the target genes using a LightCycler® 480 II (Roche, Germany) instrument. Results were obtained by using 2^−ΔΔCp^ calculation method. The primer sequences are available in Supplementary Table 2.

### ELISA

Serum IL-1β was measured from serum samples with a mouse specific IL-1β ELISA kit purchased from Thermo Fisher Scientific (Basingstoke, Hampshire, UK) according to the manufacturer’s protocol. Briefly, after initial washing steps, 100 μL of blanks, standards, and samples were loaded and were incubated for 2 h with biotin-labeled detection antibodies. Following a washing step, Streptavidin-HRP was loaded and incubated for 1 h and after another round of washing, substrate solution was added and incubated for 20 min; then, stop solution was loaded and the colorimetric reaction was measured at 450 nm with a ThermoFisher MultiSkan GO spectrophotometer (Waltham, MA, USA).

### Statistical analysis

All values are presented as mean ± standard error of mean (SEM). *P* < 0.05 was considered statistically significant. Normal distribution of data was tested by the Shapiro–Wilk normality test. One-way ANOVA followed by Fischer’s LSD post hoc test or Kruskal–Wallis test followed by uncorrected Dunn’s post hoc test were used for multiple comparison analyses. ROUT analysis was performed to identify outliers, with Q value = 1%. The statistical analyses were performed with the GraphPad Prism (version 8.0.1.) software.

## Results

### CDAA diet induces key histopathologic features of NASH

**Fig. 1:**
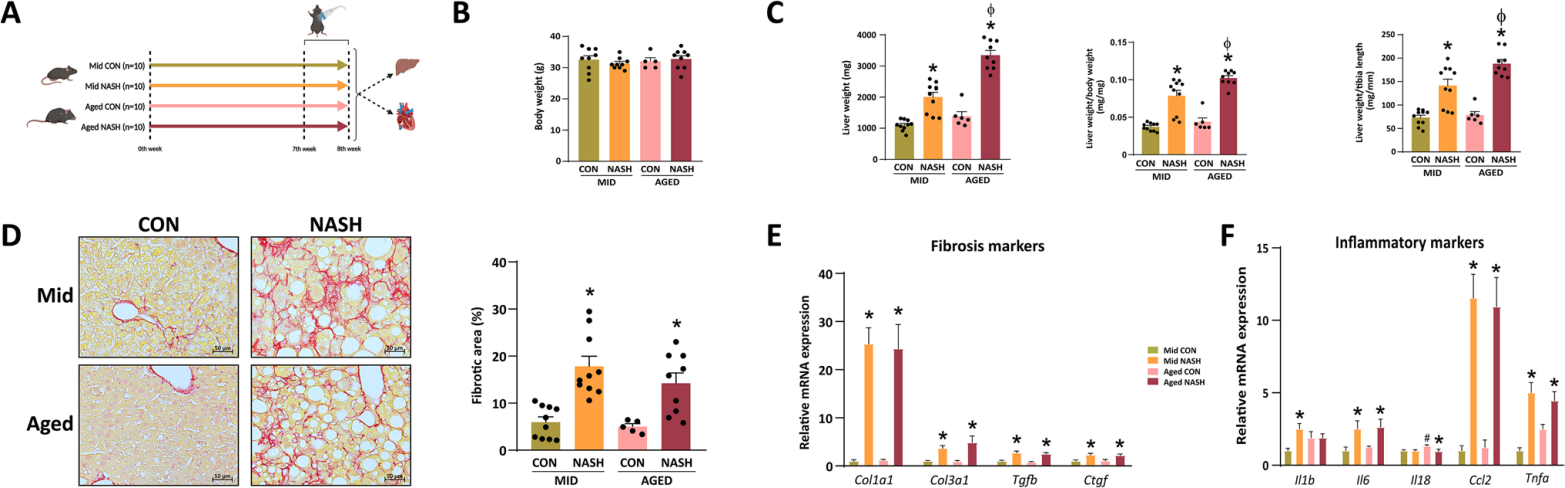
CDAA diet induces key hepatic features of NASH. Study design (**A**). Body and liver weight, (*n* = 5–10) (**B**, **C**). Picrosirius red staining and its macroscopic quantification (*n* = 5–10). The representative images were capture at × 20 magnification (**D**). Quantitative real-time PCR of pro-fibrotic and pro-inflammatory genes (*n* = 4–6) (**E**, **F**). CON, control diet; NASH, non-alcoholic steatohepatitis; MID, middle aged. One-way ANOVA followed by Fischer’s LSD post hoc test or Kruskal–Wallis test followed by uncorrected Dunn’s post hoc test, *P* < 0.05 was considered significant difference, * shows difference between age-matched cohorts, # shows difference between control animals, ϕ shows difference between animals with NASH

NASH is characterized by hepatic steatosis with displaced nuclei, extensive inflammation, and fibrosis [15]. In our previous studies, we already showed that 8 weeks of feeding with CDAA diet effectively induces classical histologic signs of NASH [14, 16] (Fig. 1A); nonetheless, we performed histologic and molecular analyses to evidence the development of NASH in the present cohort. The rationale for choice of model was that we wished to investigate the effects of NASH on the cardiovascular system without the systemic burden of obesity, insulin resistance, hypertension, and dyslipidemia. As such, our animals did not differ in body weight (Fig. 1B), but the liver weight of animals with NASH was significantly elevated, especially in the aged group (Fig. 1C).

Histologic analysis of the liver with picrosirius-red staining evidenced liver fibrosis in animals fed with CDAA diet. Subsequent quantification of the fibrosis revealed significant fibrosis in both middle-aged and aged mice fed with CDAA (Fig. 1D).

Quantitative real time PCR measurement showed elevated expression of pro-fibrotic genes, such as *Col1a1*, *Col3a1*, *Tgfb*, and *Ctgf* (Fig. 1E). Major inflammatory markers were examined as well. The gene expression of *Il6*, *Ccl2*, and *Tnfa* was significantly increased compared to their age-matched controls (Fig. 1F). *Ccl2* gene expression was the highest, thus supporting its relevance in the pathomechanism of NASH [17]. The hepatic expression of *Il1b* was increased only in middle aged mice with NASH (Fig. 1F).

In summary, CDAA diet induces NASH in both middle-aged and aged animals.

### Strain rate analysis is able to identify diastolic dysfunction, while conventional echocardiography is not

In this section, we aimed to evaluate the cardiac geometry and function with both conventional and with 2D-speckle tracking echocardiography.

**Fig. 2:**
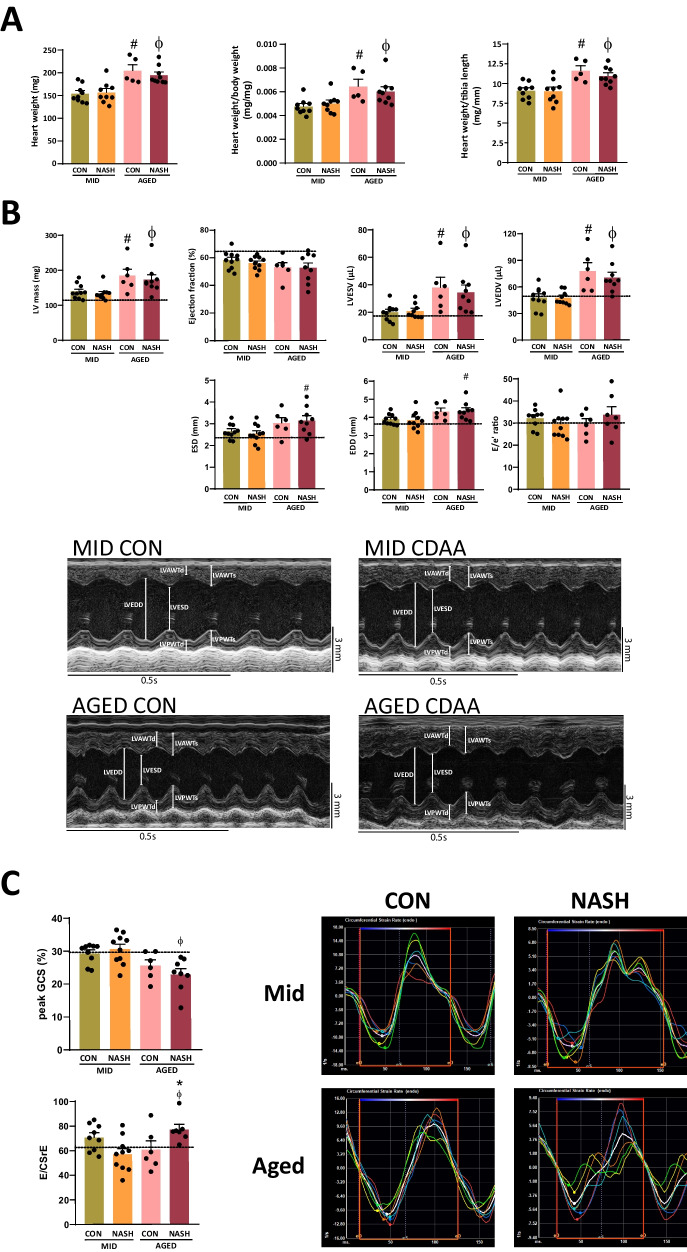
Conventional and two-dimensional speckle tracking echocardiography. Heart weight (*n* = 5–10) (**A**). Bar graphs of conventional echocardiographic parameters with representative images of parasternal short axis M-mode (*n* = 5–10) (**B**). Bar graphs of two-dimensional speckle tracking echocardiographic parameters with representative images of strain rate analysis (*n* = 5–10) (**C**). The dotted lines represent average values of young animals. CON, control diet; NASH, non-alcoholic steatohepatitis; MID, middle aged; LV, left ventricle; LVESV, left ventricular end-systolic volume; LVEDV, left ventricular end-diastolic volume; ESD, end-systolic diameter, EDD, end-diastolic diameter; GCS, global circumferential strain; CSrE, early diastolic strain rate of circumferential fibers. One-way ANOVA followed by Fischer’s LSD post hoc test or Kruskal–Wallis test followed by uncorrected Dunn’s post hoc test, *P* < 0.05 was considered significant difference, * shows difference between age-matched cohorts, # show difference between control animals, ϕ shows difference between animals with NASH

Aged mice had greater cardiac weight (Fig. 2A). Additionally, conventional echocardiographic analysis of left ventricular mass showed increased chamber weight in aged animals (Fig. 2B). Analysis of parasternal long-axis (PSLAX) images showed declining, but still normal ejection fraction in middle-aged mice with NASH and in both aged groups (Fig. 2B). PSLAX view of the left ventricle revealed increased end-systolic- and end-diastolic volumes (LVESV, LVEDV) in aged animals with NASH (Fig. 2B). Parasternal short-axis (PSAX) images showed increased left ventricular end-systolic- and end-diastolic diameter (ESD, EDD) in aged animals with NASH. Intraventricular pressure was assessed by the ratio of early mitral inflow velocity-to-early diastolic mitral annulus velocity (*E*/*e*′). This conventional parameter of diastolic function did not reveal sign of deterioration (Fig. 2B).

Deterioration of myocardial torsion and deformation could be an early sign of cardiac dysfunction. As such, a more sensitive method, two-dimensional speckle tracking echocardiography was performed. Peak global circumferential strain (GCS) confirmed the systolic decline evidenced by conventional echocardiography (Fig. 2C). A relatively novel parameter is the ratio of early diastolic transmitral velocity (*E*) and early diastolic strain rate (SrE). Strain rate analysis measures the torsion of cardiac fibers in time. In our model, we observed a significant increase in E/CSrE in aged mice with NASH, suggesting an increase in diastolic filling pressure (Fig. 2C).

In summary, we report age-dependent decline of systolic function, increased LV mass, and diastolic dysfunction in aged animals with NASH.

### NASH triggers cardiac hypertrophy, fibrosis, and inflammation

**Fig. 3:**
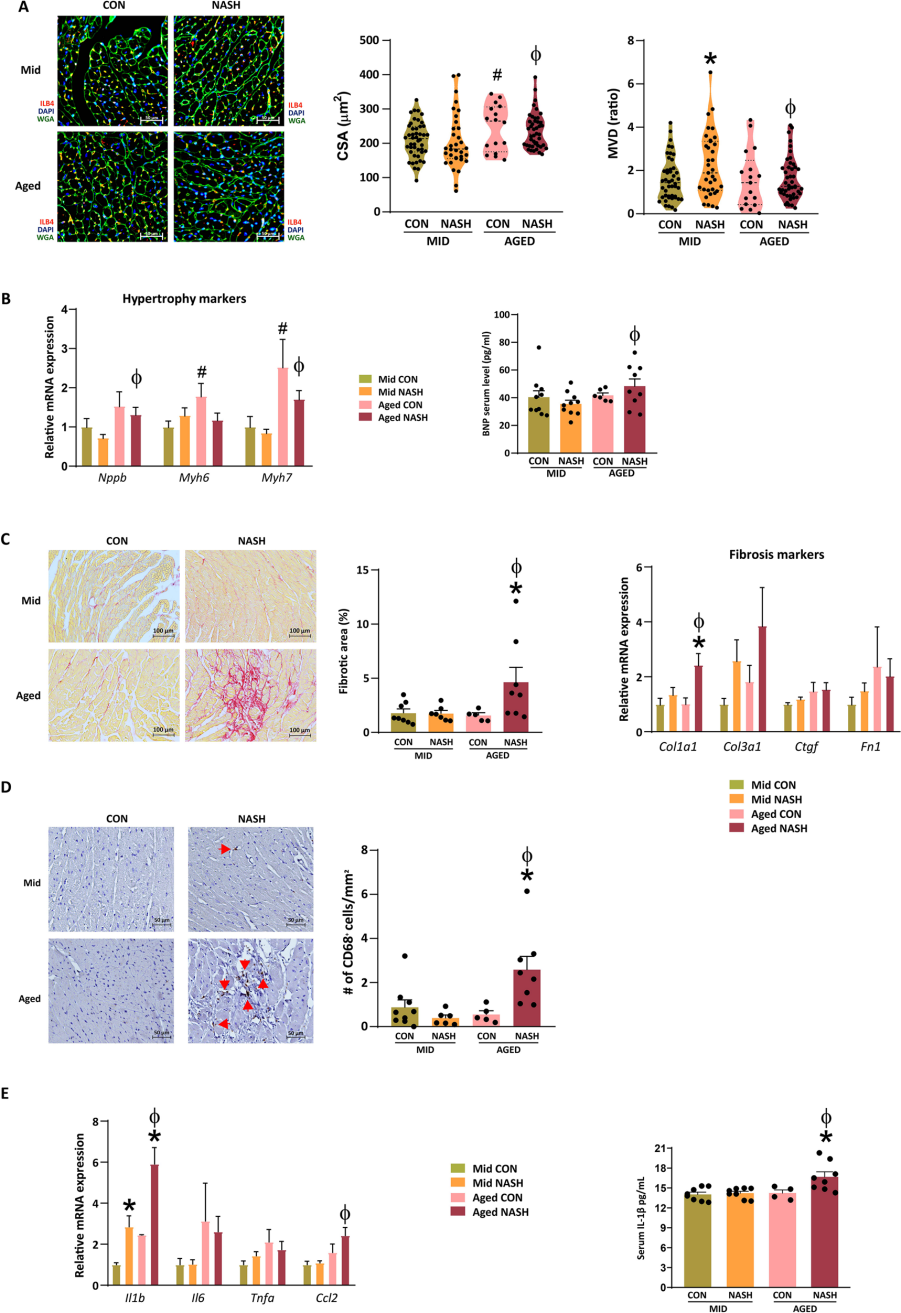
Characterization of cardiac morphology in mice with NASH. Lectin histochemistry (*n* = 5–10). Blue shows nuclei, red shows cardiac endothelial cells, and green shows the cell membrane of cardiomyocytes. Quantification of cross-sectional area and microvascular density (**A**). Bar graphs of cardiac hypertrophy markers (*n* = 4–6) and BNP serum level (*n* = 6–10) (**B**). Cardiac picrosirius red staining and its macroscopic quantification. Bar graphs of pro-fibrotic genes (*n* = 5–10) (C). Immunostaining of CD68^+^ macrophages and its quantification (*n* = 5–10) (**D**). Gene expression of pro-inflammatory cytokines in the heart and serum IL-1β level (*n* = 4–8) (**E**). CON, control diet; NASH, non-alcoholic steatohepatitis; MID, middle aged; BNP, b-type natriuretic peptide. One-way ANOVA followed by Fischer’s LSD post hoc test or Kruskal–Wallis test followed by uncorrected Dunn’s post hoc test, * shows difference between age-matched cohorts, # shows difference between control animals, ϕ shows difference between animals with NASH

Next, we aimed to evaluate the effects of NASH on cardiac morphology. First, we performed lectin histochemistry to assess cardiac remodeling. Aged animals with NASH had increased cross-sectional area (CSA) of endocardial myofibers compared to middle-aged animals with NASH. Aged animals with NASH were characterized with decreased capillary density (Fig. 3A). These data suggest a failure of the capillary system to cope with cardiomyocyte hypertrophy in aged mice with NASH. Gene expression analysis showed that *Myh6* and *Myh7* expression increased in aged control animals (Fig. 3B). The gene expression level and serum level of B-type natriuretic peptide (BNP) were only elevated in aged animals with NASH (Fig. 3B).

Furthermore, aged animals with NASH showed significant cardiac fibrosis as well. Among the investigated pro-fibrotic genes, *Col1a1* was significant elevated in the cardiac samples of aged animals with NASH (Fig. 3C).

Cardiac macrophages were shown to contribute to the pathomechanism of diastolic dysfunction and HFpEF [18]. Immunohistochemistry was performed to determine whether CD68^+^ monocytes/macrophages infiltrate into the heart in this model. Quantification of these cells showed elevated CD68^+^ cell count only in aged mice with NASH (Fig. 3D). Quantitative real-time PCR revealed elevated expression of *Il1b* and *Ccl2* in cardiac samples of aged NASH cohort, suggesting a more pronounced inflammatory environment in the heart of aged animals with NASH (Fig. 3E). Lastly, we measured the serum level of IL-1β, and we found that aged mice with NASH had significantly higher level of this pro-inflammatory cytokine (Fig. 3E).

## Discussion

In this paper, we report that there is a link between NASH and diastolic dysfunction, which is a major hallmark of HFpEF. Both diseases are increasing concerns for healthcare professionals for several reasons: both diseases comprise a heterogenous population with a combination of a wide-variety of co-morbidities, little-to-no effective pharmacotherapy, difficulty of diagnosis, and most importantly with ever-growing number of patients. Consequently, understanding the pathomechanism and the potential link between the two diseases is crucial to develop pharmacotherapy capable of decreasing mortality and morbidity.

Within the field of cardiology, treatment of HFpEF is still an unmet need. One difficulty results from the heterogeneity of the patient population [19], thus demanding a distinctive subgroup specific therapy. As such several research groups aimed to cluster these wide-variety of HFpEF patients by phenomapping [20–24]. Several clusters were suggested based on pathophysiologic, clinical, and biologic findings. Shah et al., for example, proposed 7 phenotypes: (i) cardiometabolic, (ii) coronary artery disease-associated and (iii) atrial fibrillation, (iv) right heart failure-dominant, (v) hypertrophic, (vi) valvular and (vii) restrictive cardiomyopathy-related ones [20]. Cardiometabolic phenotype of HFpEF comprises of hypertension, obesity, insulin resistance, chronic kidney disease, and metabolic syndrome. As such, the question arises that whether NASH, a chronic meta-inflammatory liver disease, contributes to the development of the cardiometabolic HFpEF.

Clinical studies suggested a possible link between NAFLD and diastolic dysfunction, which is the most relevant functional abnormality of HFpEF. In 2006, it was reported for the first time that NAFLD might give rise to cardiac hypertrophy and diastolic dysfunction. They showed increased posterior, septal wall thickness and left ventricular mass, suggesting enlarged heart size, in patients with NAFLD. Furthermore, grade I—diastolic dysfunction was established in patients with NAFLD [25]. Fatty liver is the benign stage of NAFLD; nonetheless, cardiac deterioration has already developed at such an early phase of this progressive chronic disease. NAFLD patients in this clinical study had increased BMI (on average 31.4 kg/m^2^); however, the authors did not observe correlation between BMI and E/A (*r* = 0.13, *p* = 0.6), an indirect marker of intraventricular pressure, or between BMI and left ventricular mass index (*r* = 0.06, *p* = 0.8), suggesting that besides the systemic burden of obesity, other factors may fuel the deterioration of cardiac function. The question arises whether NAFLD or its subsequent stage, NASH, is a contributing factor. A paper published in 2009 reported that patients with primary hypertension had greater prevalence of diastolic dysfunction if NAFLD was present [26]. These results suggest that diastolic dysfunction develops due to multiple insults, and it is likely that NAFLD-derived cardiometabolic inflammation is a major driver. The aforementioned two studies had a relatively low participant number though. However, VanWagner et al. investigated close to 3000 patients of the CARDIA study to assess a potential link between NAFLD and heart failure. They found that patients with NAFLD had higher left ventricular filling pressure and worse myocardial fiber strain. Furthermore, NAFLD was associated with subclinical cardiac remodeling [5]. The higher number of participants further increases the evidence for this hepato-cardiac link. The extent of cardiac remodeling, epicardial fat volume, and diastolic dysfunction is greatly associated with liver disease and/or fibrosis severity [27–31]. Additionally, NAFLD is associated with coronary artery disease [31–34]; not surprisingly, since dyslipidemia is key risk factor for NAFLD, consequently, one of the main outcomes for patients with NAFLD is cardiovascular death.

Participants of the previously cited studies have 1 or more co-morbidities besides NAFLD; thus, it is important to delineate whether NAFLD or NASH is a mere facilitator of the cardiac remodeling and dysfunction promoting effect of other risk factors (e.g., obesity, hypertension) or whether it is a sole driver of the cardiometabolic phenotype of HFpEF. As such, we aimed to investigate whether diastolic dysfunction develops in a preclinical model of NASH, in which obesity, hypertension, and insulin resistance do not develop [35]. Although the CDAA diet is high in cholesterol (2%), male mice did not develop hypercholesterolemia nor hypertriglyceridemia in a previous study of ours [14]; thus, the burden of dyslipidemia can also be excluded.

We report that 8 weeks of CDAA diet induced key features of NASH, such as steatosis, inflammation, and fibrosis, in both middle-aged and aged C57Bl/6 J mice (Fig. 1). Elderly animals were characterized with greater heart weight (Fig. 2), translating to the findings of NAFLD patients undergo cardiac remodeling and develop cardiac hypertrophy resulting increased left ventricular muscle mass [5].

Conventional echocardiography supports the aforementioned increased left ventricular muscle mass and cardiac remodeling by evidencing increased left ventricular end-systolic and end-diastolic volumes in aged animals (Fig. 2B). Furthermore, aged mice with NASH had increased intraventricular diameter (Fig. 2B). Pulse wave and tissue Doppler did not evidence deterioration in diastolic indices. A previous study suggested that *E*/*e*′, an indirect marker of left ventricular pressure, is less reliable in scenarios where the ejection fraction is preserved [36]. Alternatively, two-dimensional speckle tracking echocardiography was shown to be able to reveal even subtle myocardial deterioration before clinical dysfunction manifests [37, 38]. Early diastolic strain rate was shown to evidence subclinical diastolic deterioration in patients with aortic stenosis [39]. Consequently, we perform strain analysis to determine whether myocardial torsion and/or strain is affected by our disease model. Peak global circumferential strain significantly decreased in aged mice with NASH (Fig. 2C**)**. In human studies, global longitudinal and radial strain deterioration was reported so far [4, 40]. Regarding the diastolic function, we report that the ratio of early trans mitral flow velocity-to-early diastolic strain rate of the circumferential fibers increased in aged mice with NASH (Fig. 2C), suggesting an elevated left ventricular pressure. This finding proves that NASH in aged lean animals deleteriously affects myocardial relaxation. To further support the predictive value of speckle tracking echocardiography in patients with NAFLD, a clinical trial will shortly begin (NCT05790057).

After establishing the impact of NASH on cardiac function, we aimed to characterize the morphology and the potential remodeling of the heart. First, lectin histochemistry was performed with wheat germ agglutinin (marker of cardiac cell membrane) and isolectin B4 (marker of cardiac endothelial cells) (Fig. 3A). Cross-sectional area (CSA) of cardiomyocytes increased in both aged cohorts. However, aged animals with NASH showed decreased microvascular density compared to middle-aged mice with NASH. This finding suggests that the capacity of the capillary system to cope with myocardial hypertrophy is exhausted in aged mice with NASH, supporting the hypothesis of endothelial microvascular dysfunction in HFpEF [41]. Furthermore, it has been shown that patients with HFpEF have decreased microvascular density [42]. Prevalence of left ventricular hypertrophy increases with age [43]. Age-related cardiac remodeling is usually fueled by increased afterload [44], i.e., vascular hypertrophy. The vascular system, as well, undergoes remodeling with age, characterized by increased media-to-lumen ratio, increased stiffness and inflammation [45]. Furthermore, speckle tracking studies revealed that the elders have diminished “untwisting” of myocardial fibers [46] and global circumferential strain, which is presumed to be caused by myocardial interstitial fibrosis [47].

Cardiac aging further limits the already limited regenerative potential of the heart [48], which may contribute to pathologic remodeling of the heart by resulting a tissue that is non-compliant and inflexible to insults.

Aging hearts have a distinctive metabolic profile compared to an adult heart. Lipid oxidation contributes to a lower extent to produce energy in aged cardiomyocytes [49], while anaerobic glycolysis dominates over glucose oxidation [50] promoting cardiac hypertrophy and systolic dysfunction [51]. Several secreted pro-inflammatory and non-inflammatory molecules might contribute to cardiac aging, such as interleukin-1β or interleukin-6 and insulin-like growth factors, by promoting atherosclerosis and insulin resistance [52, 53], respectively.

Although hypertrophy, in our model, can be attributed to aging, diastolic dysfunction is likely to be the consequence of the combination of aging and prolonged low-grade inflammatory signaling in the heart. Mouse models of metabolic syndrome are characterized by Th1 type inflammation resulting myocardial stiffness driven by fibrogenesis [54]. IL-1R signaling was shown to deleteriously affect diastolic function by changing the ratio of expression of phospholamban and sarcoplasmic Ca^2+^ ATPase [55]. In a rat model, IL-6 was shown to promote cardiac remodeling and diastolic dysfunction [56]. CCL2 contributes to cardiac dysfunction via TLR4 [57]. In addition, several cross-sectional observational studies showed that patients with diastolic dysfunction and/or diabetes were associated with increased IL-6, IL-8, and CCL2 [58, 59].

Previously, it was shown that CCL2 is the main driver of myeloid cell infiltration during NAFLD/NASH [17]. Our results of increased cardiac *Ccl2* expression coincides with increased CD68^+^ cell infiltration in aged mice with NASH. It was shown that increased number of CD68^+^ macrophages promotes fibrosis and inflammation [60], by secreting interleukin-1β, which was shown to uncouple β-adrenergic receptors from l-type Ca^2+^ channels [61], disturb cellular energetics [62], and deteriorate cardiac Ca^2+^ homeostasis [63], through modulating phospholamban and SERCA expression [63].

Cardiac aging is often similar to a “hypothyroid state,” a condition when downregulation of the thyroid hormone receptor β1 results in differential expression of myosin heavy chain isoforms [64]. One of the first steps of cardiac remodeling is the upregulation of contractile myofibrils resulting in hypertrophy and cardiac dysfunction. Additionally, hypothyroidism is often associated with NASH [65].

Next, we performed qRT-PCR to assess gene expression of cardiac hypertrophy markers. Genes of *Myh6* and *Myh7* were increased in CON diet–fed aged animals (Fig. 3B). The expression of *Nppb* was increased in aged mice with NASH only. This finding was supported by measuring serum level of BNP (Fig. 3B). The guideline of the European Society of Cardiology 2021 already includes elevated natriuretic peptide levels as a key diagnostic criterion for HFpEF. However, it also highlights that 20% of patients with HFpEF have normal or low levels of natriuretic peptides. Salah et al. proposed three NAFLD-driven phenotypes of HFpEF: obstructive, meta-inflammatory, and cirrhotic [66]. They argue that in the obstructive HFpEF phenotype, preload reserve depletion causes decreased intracardiac filling pressures resulting in low levels of natriuretic peptides [67, 68]. In our model, we report increased filling pressure (E/CSrE) and increased serum BNP level. Therefore, the suggested hepatic sinusoidal obstruction can be disregarded in our model. In the meta-inflammatory phenotype, the common feature of both NALFD and HFpEF is inflammation. In our model, we report elevated intracardiac CD68^+^ monocyte/macrophage count in the aged cohort with NASH (Fig. 3D). Some studies suggested that NAFLD might be a contributor of atrial fibrillation [69], and this finding was associated with macrophage-derived IL-1β [70]. Additionally, the gene expression of *Il1b* and *Ccl2* was increased in the same group (Fig. 3E). ELISA measurement of serum IL-1β revealed that aged mice with NASH had higher levels, but the overall level was low compared to a major acute injury [71], further supporting the role of chronic low-grade inflammation.

Similarly to our previous publication [16], we established that IL-1β is relevant not only for the hepatic pathophysiology of NASH, but it is highly expressed in cardiovascular system as well. This highlights that inflammation could be an important therapeutical target, which is further supported by the CANTOS trial [72]. Most drug trials that aimed to treat NASH targeted metabolic processes, while clinical trials targeting inflammation in NASH are low in number [73]. Additionally, we highlight that not only soluble mediators (i.e., IL-1β, CCL2) are relevant drug targets within this hepato-cardiac axis, but cellular culprits can also be identified, such as intracardiac infiltrating monocytes/macrophages [18].

We found that only aged mice with NASH developed significant cardiac fibrosis (Fig. 3B). In addition, collagen type I was significantly overexpressed in the heart of aged mice with NASH (Fig. 3B). Metabolic, hemodynamic, and immunologic stress facilitates myocardial fibrosis generation in HFpEF [74]. Accordingly, myocardial fibrosis was shown to be a major determinant in all-cause mortality in HFpEF patients [75].

In conclusion, we highlight that more specific methods are needed to evidence subtle myocardial deterioration in HFpEF, and show that speckle tracking echocardiography is capable to reveal such subtle changes, allowing early diagnosis of this population. Furthermore, we have found that NASH without any systemic burden is per se a contributing factor of diastolic dysfunction and/or HFpEF upon aging.

## Limitations

Although echocardiographic analyses are considered important measurements to assess cardiac function, pressure–volume loop analysis would have shown the exact intraventricular conditions.

### Supplementary Information

Below is the link to the electronic supplementary material.

Supplementary file1 (DOCX 15 KB)

Supplementary file2 (DOCX 16 KB)

## Data Availability

The data presented in the present study are available from the corresponding author on reasonable request.
