# Supplementary material for: NASH triggers cardiometabolic HFpEF in aging mice
Source: GeroScience. 2024 Apr 17;46(5):4517–31. doi: 10.1007/s11357-024-01153-9 (PMC11336017; doi:10.1007/s11357-024-01153-9)
Supplement: Supplementary file 1 — Supplementary file1 (DOCX 15 KB) [file 11357_2024_1153_MOESM1_ESM.docx]

**NASH triggers cardiometabolic HFpEF in aging mice**

**Dániel Kucsera, PharmD^1,2,3^, Mihály Ruppert, MD, PhD^4^, Nabil V. Sayour, MD^1,2,3^, Viktória E. Tóth, PharmD, PhD^1,2,3^, Tamás Kovács, MSc^1,2,3^, Zsófia Onódi, MD, PhD^1,2,3^, Alexandra Fábián, MD^4^, Attila Kovács, MD, PhD ^4^, Tamás Radovits, MD, PhD^4^, Béla Merkely, MD, PhD^4^, Pál Pacher, MD, PhD^5^, Péter Ferdinandy, MD, PhD^1,6^, Zoltán V. Varga, MD, PhD^1,2,3^**

^1^Department of Pharmacology and Pharmacotherapy, Semmelweis University, Budapest, Hungary;

^2^HCEMM-SU Cardiometabolic Immunology Research Group, Budapest, Hungary;

^3^MTA-SE Momentum Cardio-Oncology and Cardioimmunology Research Group, Budapest, Hungary;

^4^Heart and Vascular Center, Semmelweis University, Budapest, Hungary;

^5^Laboratory of Cardiovascular Physiology and Tissue Injury, National Institutes of Health/National Institute on Alcohol Abuse and Alcoholism, Bethesda, Maryland, USA;

^6^Pharmahungary Group, Szeged, Hungary.

Corresponding author: Zoltán V. Varga (varga.zoltan@med.semmelweis-univ.hu)

| Antibodies/lectins | Producer | Cat. number | Country of origin | Dilution | Comments |
| --- | --- | --- | --- | --- | --- |
| isolectin B4 | Sigma Aldrich | L4895 | USA | 1:50 | marker of endothelial cells |
| wheat germ agglutinin | Invitrogen | L32473 | USA | 1:50 | marker of myocardial cell membrane |
| DAPI | Cell Signaling Technology | 4083S | USA | 1:1000 | marker of nucleus |
| Iba1 | Wako Pure Chemical Industries | 019-19741 | Japan | 1:2000 | marker of macrophages |
| anti-rabbit HRP | Cell Signaling Technology | 8114S | USA | - | secondary antibody |
