# Supplementary material for: NASH triggers cardiometabolic HFpEF in aging mice
Source: GeroScience. 2024 Apr 17;46(5):4517–31. doi: 10.1007/s11357-024-01153-9 (PMC11336017; doi:10.1007/s11357-024-01153-9)
Supplement: Supplementary file 2 — Supplementary file2 (DOCX 16 KB) [file 11357_2024_1153_MOESM2_ESM.docx]

**NASH triggers cardiometabolic HFpEF in aging mice**

**Dániel Kucsera, PharmD^1,2,3^, Mihály Ruppert, MD, PhD^4^, Nabil V. Sayour, MD^1,2,3^, Viktória E. Tóth, PharmD, PhD^1,2,3^, Tamás Kovács, MSc^1,2,3^, Zsófia Onódi, MD, PhD^1,2,3^, Alexandra Fábián, MD^4^, Attila Kovács, MD, PhD ^4^, Tamás Radovits, MD, PhD^4^, Béla Merkely, MD, PhD^4^, Pál Pacher, MD, PhD^5^, Péter Ferdinandy, MD, PhD^1,6^, Zoltán V. Varga, MD, PhD^1,2,3^**

^1^Department of Pharmacology and Pharmacotherapy, Semmelweis University, Budapest, Hungary;

^2^HCEMM-SU Cardiometabolic Immunology Research Group, Budapest, Hungary;

^3^MTA-SE Momentum Cardio-Oncology and Cardioimmunology Research Group, Budapest, Hungary;

^4^Heart and Vascular Center, Semmelweis University, Budapest, Hungary;

^5^Laboratory of Cardiovascular Physiology and Tissue Injury, National Institutes of Health/National Institute on Alcohol Abuse and Alcoholism, Bethesda, Maryland, USA;

^6^Pharmahungary Group, Szeged, Hungary.

Corresponding author: Zoltán V. Varga (varga.zoltan@med.semmelweis-univ.hu)

| **Gene name** | Accession number | Forward primer | Reverse primer | Product size (bp) |
| --- | --- | --- | --- | --- |
| *Ccl2* | NM_011333.3 | ACCTGCTGCTACTCATTCACC | CTCTTGAGCTTGGTGACAAAAACTA | 119 |
| *Col1a1* | NM_007742.4 | TCTCCACTCTTCTAGTTCCT | TTGGGTCATTTCCACATGC | 266 |
| *Col3a1* | NM_009930.2 | GCTCGAGGCAATGATGGT | ACCCTGCAGGTCCAACTTC | 118 |
| *Ctgf* | NM_010217.2 | AGCGGTGAGTCCTTCCAAAG | TTCCAGTCGGTAGGCAGCTA | 222 |
| *Il1b* | NM_008361.4 | GCACTACAGGCTCCGAGATGAAC | TTGTCGTTGCTTGGTTCTCCTTGT | 147 |
| *Il6* | NM_031168.2 | CGGCCTTCCCTACTTCACAA | TTGCCATTGCACAACTCTTTTC | 151 |
| *Il18* | NM_008360.2 | CAGGCCTGACATCTTCTGCAA | TCTGACATGGCAGCCATTGT | 105 |
| *Myh6* | NM_001164171.1 | CTCTGGATTGGTCTCCCAGC | GTCATTCTGTCACTCAAACTCTGG | 150 |
| *Myh7* | NM_080728.3 | GCCTCAGCAGAGGAGTACAG | ATGGCTGAGCCTTGGATTCTC | 86 |
| *Nppb* | NM_008726.6 | TTTGGGCTGTAACGCACTGAA | TGTGGCAAGTTTGTGCTCCA | 219 |
| *Tgfb* | NM_021578.2 | GACCGCAACAACGCAATCTA | ACCAAGGTAACGCCAGGAAT | 207 |
| *Tnfa* | NM_013693.3 | TACTGAACTTCGGGGTGATTGGTCC | CAGCCTTGTCCCTTGAAGAGAACC | 295 |
